# Medicine

**Published:** 1912-03

**Authors:** Carey Coombs


### Progress of the Medical Sciences.

### MEDICINE.

**Pericarditis.**—At this time of day the statement that pericardial inflammation is always due to bacterial infection will not provoke opposition or even comment, and most persons will be willing to go further and subscribe to a belief in the infection being blood-borne as a rule. Chatard's[1] careful investigation of the Johns Hopkins Hospital records of *pneumococcal pericarditis* shows that even when lobar pneumonia and pericardial infection co-exist the anatomical position of the pulmonary lesion is in a majority of cases remote from the pericardium; so that it is most likely that the pneumococci reach the pericardial sac through the blood-stream in the same way that they invade joints and other tissues (probably including the lung itself).

The clinical importance of pericarditis is threefold. First, the appearance of definite evidence of pericardial implication in rheumatic carditis proves that the heart is severely attacked; second, the formation of adhesions may constitute a serious addition to the burden which the heart (injured as it always is in respect of the muscle and the valves) has to bear; third, in some forms of pericarditis there is danger of massive or purulent effusion. It is therefore important to arrive early at a knowledge of the state of the pericardium in any case of cardiac inflammation, to discover the presence of adhesions or effusion, and to treat these if possible and necessary.

One sign of acute pericarditis to which Wynter[2] has drawn attention is *limitation of diaphragmatic movement.* The clinical evidences of this are immobility and even retraction of the epigastrium, and fixation of the diaphragm as demonstrated by skiagraphy. Edwards[3] also records a case of pericarditis with abdominal tenderness and rigidity. My own attention was called to the frequency with which children with rheumatic carditis complain of epigastric pain by Dr. Reginald Miller, who suggested that this might be due to extension of the infective process from the pericardium to the sub-diaphragmatic peritoneum, and I have since noted several facts which support this view. In one case, that of a girl whose heart had been attacked by rheumatism which was quiescent at the time of examination, a very distinct rub was heard over

[1] Chatard, *Johns Hopkins Hosp. Rep.*, 1910, xv. 155.
[2] Wynter, *Clin. J.*, 1911, xxxviii. 97.
[3] Edwards, *J. Am. M. Ass.*, 1911, lvi. 1784.

the apex of the epigastrium synchronous with the breathing movements; and more recently Dr. Scott Williamson showed me flakes of fibrin on the anterior surface of the liver at an autopsy on a child who had died of acute rheumatic carditis, with widespread inflammation of the pericardium. Be this as it may, it is beyond all doubt important to recognise epigastric immobility as an early sign of pericarditis.

When we come to a consideration of the diagnosis of *pericardial effusion* we are confronted by a confused tangle of conflicting statements. Fortunately a recent discussion at the Royal Society of Medicine[1] has done much to separate fact from theory, and thus to provide a sounder basis for bedside investigation. The chief points made were that bulky pericardial effusion almost never occurs in rheumatic carditis, and that most of the signs hitherto supposed to indicate the presence of such an effusion are in reality due to dilatation of the heart, the consequence of the myocardial changes which are invariably present in acute rheumatic carditis. This latter statement applies with special force to the "pear-shaped" dulness which has been regarded as a sign of effusion. This change from the normal area is due to extension of dulness in three directions: upwards and to the left, to the right in the fourth and fifth right interspaces, and to the left at the left base. Comparison of clinical and pathological data proves that these three expansions of the cardiac dulness are due not to intrapericardial exudation but to dilatation of the left auricle, the right auricle, and the left ventricle respectively. Again, the cardiac sounds may, and often do, become indistinct in carditis as a direct result of muscular enfeeblement. It is no use to depend on disappearance of friction sounds, as even in the presence of a large effusion a rub may still be heard at the base. Hirtz[2] describes two cases of effusion in which the patient found the knee-elbow position the only comfortable attitude; but, as Miller[3] points out, children with "dry" pericarditis often lie on their faces to gain relief from pain. The "pulsus paradoxus," which Jacob and Chavigny[4] regard as the most reliable sign of effusion, was rejected by the chief speakers at the Royal Society discussion as quite uncertain.

What, then, remains? Ogle laid considerable emphasis on the value of non-pulsatile distension of the jugular veins, and West said that in his opinion extension of the area of dulness downwards and to the left, *well beyond the point of maximum impulse*, was the one reliable sign. Examination with the

[1] *Proc. Roy. Soc. Med.*, 1909–10, iii. (Med. Sect.) 55, 77.
[2] Hirtz, *Nord Méd.*, 1911, June 1st.
[3] Miller, *Medical Diseases of Children*, 1911, p. 375.
[4] Jacob and Chavigny, *Rev. de Méd.*, May, 1911.

X-rays is sometimes impracticable, but it may give valuable help. The cardiac shadow is said to become globular in effusion, with sharp non-pulsatile edges, and Rosenfeld[1] goes so far as to say that he has seen the ordinary cardiac shadow, triangular and pulsating, in the midst of such a globular area.

The fact is that it is very seldom indeed that fluid is poured out into the pericardial sac so freely as to embarrass the heart or to render paracentesis or drainage necessary. Even if it is clear that the pericardial sac should be opened, opinions differ as to the best method. The figures collected by Venus[2] go to show that incision, with resection of rib and free drainage, gives the highest percentage of recoveries. Jacob and Chavigny[3] go so far as to advise pericardiotomy without preliminary puncture; but we do not anticipate wholesale conversion of the profession to their view. Probably the majority will still prefer paracentesis, at least as a preliminary and exploratory measure. Ortner[4] advises that puncture be made in the area of dulness to which West has attributed so much diagnostic importance—that which lies to the left of the point of maximum impulse—with Curschmann's flat trocar and cannula directed towards the apex beat. If fluid be found, its withdrawal should be effected gradually by syphon action.

*Adhesion* is a much commoner result of pericardial inflammation than effusion, but its exact clinical importance is difficult to gauge. Fenton's[5] conclusions are worthy of special attention, as they are founded on a wide examination of autopsy records. He finds two types of pericardial adhesion: the first is that of childhood, in which valves are also diseased and the heart hypertrophied, the whole group of changes being due to rheumatic invasion of all the cardiac tissues simultaneously. In such cases there are adhesions without as well as within the pericardial sac, and their effect upon the efficiency of the heart is appreciable and sometimes serious. The other type of adhesion is that of adult life; it is not rheumatic in origin, it is intrapericardial only, and its clinical importance is practically *nil*. In the first class of case the adhesions exercise their deleterious influence on the efficiency of the heart rather by preventing recovery from the acute stage of carditis than by adding to the permanent burden of extra work which the heart is sentenced to bear in the form of valvular cicatrisations.

The diagnosis of pericardial adhesion is a matter of peculiar difficulty. The question to be answered at the bedside is, "Are

[1] Rosenfeld, *Münch. Med. Wchnschr.*, 1910, lvii. 1143.
[2] Venus, *Med. Press and Circ.*, Nov. 11th, 1908.
[3] Jacob and Chavigny, *loc. cit.*
[4] Ortner, *Deutsch. Med. Wchnschr.*, May 19th, 1910.
[5] Fenton, *Practitioner*, 1908, lxxxi. 637.

there adhesions which make the prognosis worse ?" It is easy enough to guess that in any given case of rheumatic disease of the heart there must be some degree of pericardial symphysis. The difficulty is to assess the importance of such adhesions. There is no better way of doing this than by a general weighing of the clinical evidence. As Moore [1] says, the presence of serious adhesions is to be inferred from a relative failure of recovery from acute cardiac rheumatism—that is, an abnormal severity and persistence of symptoms and signs after the patient has passed out of the years in which rheumatism is active. The inference of adhesion in such cases is materially strengthened if, at an earlier date, direct evidence of pericardial inflammation has been noted.

None of the physical signs which have been described as diagnostic of adhesion are quite reliable ; most of them are rather to be ascribed to the permanent enlargement of the heart, which is an invariable accompaniment of widespread adhesion, but which may of course be present when there is little or no adhesion. Even Broadbent's sign (systolic recession of the left lower ribs behind) may be most definitely present in cases of cardiac enlargement which autopsy proves to be independent of pericardial disease, as in Paterson's [2] case, and also in a scarcely less striking case observed by myself.

A proper appreciation of the almost insuperable difficulty of diagnosing adherent pericardium will militate against a too ready enthusiasm for the operation euphemistically called *cardiolysis*. Here is a lesion, adherent pericardium, which cannot be surely diagnosed. It is only a part, and often a relatively unimportant part, of a general disease of the heart, rheumatic pancarditis. It is not pretended that radical cure, even of the pericardial fraction of this disease, can be accomplished by operation, and yet it is proposed to dignify a clumsy attack upon a vital organ by the term "cardiolysis." It is only fair to say that some benefit appears to follow resection of parts of the præcordial thorax in cases of cardiac enlargement following rheumatic infection ; but Bewley [3] and Morison, [4] who have submitted patients to the operation, believe that this is due to provision of more room for the exaggerated movements of the heart, rather than to liberation of its action by breaking down of adhesions, and that therefore the term "thoracostomy" is more accurate.

Carey Coombs.

[1] Moore, *Lancet*, 1910, ii.

[2] Paterson, *Glasgow M. J.*, 1911, lxxvi. 24.

[3] Bewley, *Brit. M. J.*, 1910, i. 914.

[4] Morison, *Lancet*, 1908, ii. 7.

